# Transient Receptor Potential (TRP) Channels in Health and Disease

**DOI:** 10.3390/cells8050413

**Published:** 2019-05-04

**Authors:** Alexander Dietrich

**Affiliations:** Walther-Straub-Institute of Pharmacology and Toxicology, Member of the German Centre for Lung Research (DZL), Medical Faculty, LMU Munich, Nussbaumstr. 26, D-80336 Munich, Germany; Alexander.Dietrich@lrz.uni-muenchen.de

Almost 25 years ago, the first mammalian transient receptor potential (TRP) channel, now named TRPC1, was cloned and published (reviewed in [1]). Although the exact function of TRPC1 is still elusive [1], TRP channels now represent an extended family of 28 members, fulfilling multiple roles in the living organism [2]. Their identified functions include control of body temperature, transmitter release, mineral homeostasis, chemical sensing, and survival mechanisms in a challenging environment. The TRP channel superfamily covers six families: TRPC, with C for “canonical”; TRPA, with A for “ankyrin”; TRPM, with M for “melastatin”; TRPML, with ML for “mucolipidin”; TRPP, with P for “polycystin”; and TRPV, with V for “vanilloid” (see Figure 1). They all share a structure of six transmembrane (TM) regions, with a pore domain between TM5 and 6 and cytoplasmic amino- and carboxyl-termini. Functional nonselective, Ca^2+^ permeable TRP channels are tetramers, which consist of the same or different TRP monomers preferentially from the same family [3]. 

Eleven mutant TRP channels cause a spectrum of 16 human diseases, additionally emphasizing their essential role in vivo [2]. Moreover, TRP channels are important pharmacological targets for specific novel therapeutic treatment options for patients. Along these lines, specific TRP modulators have been identified in recent years and are now tested in vitro and in vivo against symptoms caused by dysfunctional TRP proteins or pathophysiological processes (such as pain, chronic inflammation, fibrosis, and edema), which occur if normal physiological responses are out of control [2,4].

Over the last few years, new findings on TRP channels confirm their exceptional function as cellular sensors and effectors. This special issue of Cells features a collection of eight reviews and seven original articles summarizing the current state-of-the-art research on TRP channels, with a focus on TRP channel activation, their physiological and pathophysiological function, and their roles as pharmacological targets for future therapeutic options. 

Returning to the roots of the mammalian TRP channel discovery, TRPC1 may preferentially work as a regulator of heterotetrameric **TRPC1/4/5** channels rather than of a homomeric TRPC1 ion channel (reviewed in [1]). Dr. Minard and colleagues present an excellent overview on the function of these heteromeric channel complexes in different tissues and pathologies, and they introduce specific small molecular modulators that are important for future research and as therapeutic options in pathophysiological processes [6].

Belonging to the same family of canonical TRPC channels, **TRPC3** controls specific functions in the cardiovascular system, the brain, the immune system, during cancer progression, and tissue remodeling, which are summarized in the comprehensive review by Drs. Tiapko and Groschner. They also present new therapeutic approaches, such as photopharmacology and optochemical genetics, to manipulate the action of TRPC3 for the intervention of its tissue-specific tasks [7].

Along the same lines, Drs. Tian and Zhu present evidence in their original article for a specific and exclusive role of **TRPC3** for the metabotropic glutamate receptor 1 (mGluR1)-mediated augmentation of slow excitatory postsynaptic currents (sEPSC) by type B γ-aminobutyric acid (GABA_B_) receptors in the Purkinje cells of the cerebellum. This molecular mechanism is essential in long-term depression, as well as synapse elimination, and may regulate motor coordination and learning [8]. 

A characteristic feature of TRPC3, TRPC6, and TRPC7 is their activation by diacylglycerol (DAG) as a product of receptor-induced phospholipase-C activity (reviewed in [9]). Recent evidence, however, suggests that **TRPC4** and **TRPC5** channels are also activated by DAG [10]. The much more complex molecular mechanism includes the C-terminal interaction with the scaffolding proteins Na^+^/H^+^ exchange regulatory factors 1 and 2 (NHERF1 and NHERF2), which dynamically regulate the DAG sensitivity of TRPC4 and TRPC5. These cellular events are summarized by Drs. Mederos y Schnitzler, Gudermann, and Storch [11].

The role of ion channels and transporters, especially that of **TRPC6** in inflammation, is the topic of a review by Dr. Ramirez and colleagues. They present an overview on TRPC6 channel activity in leucocytes, transendothelial migration, chemotaxis, phagocytosis, and cytokine release [12]. The importance of channel function is underlined by the very recent identification of a single nucleotide polymorphism (SNP) in the TRPC6 gene in patients with the autoimmune disease lupus erythematosus by the same authors [13].

**TRPA1** is the only member of the TRPA family, carrying a higher amount of ankyrin repeats (16 for human TRPA1) at the amino-terminus than other TRP proteins (usually four). Chemical modification of its cysteine residues makes TRPA1 an attractive candidate as a toxicant sensor (reviewed in [14]). Dr. Lüling and coauthors in their original article identified heat shock 70 kDa protein 6 as an effector regulated by the activation of TRPA1 by sulfur mustard (SM), a chemical warfare agent used during the civil war in Syria. The authors of this manuscript used a proteomic approach to identify differentially regulated proteins in TRPA1-expressing HEK293 and A549 cells after SM treatment. The selective TRPA1 inhibitor AP18 was used to distinguish the TRPA1-mediated effect from unspecific effects [15]. 

Moving to the TRPM family, Drs. Liu, Ong, and Ambudkar introduce an exciting role of **TRPM2** in salivary glands. Xerostomia, also known as dry mouth, is an irreversible side effect after therapeutic irradiation of head and neck cancers. TRPM2-deficient mice showed only a transient loss of salivary gland exposure with more than 60% recovery after irradiation [16]. Moreover, there is evidence for a role of this channel in inflammatory processes and inducing the autoimmune disease Sjögren’s syndrome. The involvement of TRPM2 and other TRP channels in salivary gland excretion is discussed in this comprehensive review [17]. 

Patients carrying a mutation in their **TRPM4** protein suffer from cardiac conduction disease, emphasizing TRPM4’s key role in the heart [18]. Drs. Wang, Naruse, and Takahashi highlight the functions of this channel in cardiovascular pathophysiology, e.g., ischemia-reperfusion injury causing myocardial infarction [19].

The kinase-coupled **TRPM7** channel is expressed in multiple cells of the immune system, such as lymphocytes, mast cells, neutrophils, and macrophages. Recently, it was demonstrated that the enzymatic activity of TRPM7 is required for the gut homing of intra-epithelial lymphocytes [20]. Mrs. Nadolni and Dr. Zierler shed light on how the TRPM7 channel, and/or kinase activity, is essential for pathologies, such as allergic hypersensitivity, arterial thrombosis, and graft versus host disease [21]. 

Menthol, as a cooling compound from peppermint, has been used for hundreds of years without the molecular basis of its action being revealed. Soon after cloning the eighth member—**TRPM8**—of the melastatin family of TRP channels, several laboratories have reported that natural and synthetic cooling mimetics, such as icilin, eucalyptol, and menthol, activate this channel (reviewed in [22]). Dr. Khare and colleagues now provide evidence that the application of menthol may induce a so-called “browning” effect in subcutaneous adipose tissue, although a direct involvement of TRPM8 has not been identified yet [23]. 

Two original contributions analyze the distribution of TRPV channels in human tissues using immunohistochemistry. Dr. Del Fiacco and colleagues present evidence for the expression of **TRPV1** channels in a region of the human brain, which they name Locus Karalis (Locus K). Most interestingly, TRPV1-like immunoreactivity partially overlaps with that of neuropeptides calcitonin gene-related peptide (CGRP) and substance P [24].

Drs. Rizopoulos, Papadaki-Petrou, and Assimakopoulou analyze the expression of TRPV1, TRPV2, TRPV3, and TRPV4 proteins in the mucosal epithelium of colitis ulcerosa patients in comparison to healthy volunteers. In their research, they identified a decreased expression of **TRPV1**, while **TRPV4** channels were found to be upregulated in tissues of patients. For **TRPV2** and **TRPV3**, no changes in expression levels were observed [25]. 

Many different TRP channel structures were recently resolved by cryo-electron microscopy (reviewed in [26]). In each case a large amount of pure protein material is required, which cannot be easily produced in *E. coli*, as eukaryotic post-translational processing is required for channel maturation. Therefore, another cheap eukaryotic expression system for TRP channels is presented by Dr. Zhang and colleagues. They recombinantly produced **11 human TRP members** in the yeast *Saccharomyces cerevisiae* and confirmed retained functionality for TRPM8 as the model target [27]. *S. cerevisiae* on its own also expresses a TRP channel called **TRPY1**, which is activated by increased cytosolic levels of Mn^2+^ in response to oxidative stress, as outlined in an original manuscript by Drs. Ruta, Nicolau, Popa, and Farcasanu [28].

Last but not least, Dr. Steinritz and colleagues systematically screened available literature to identify the role of TRP channels as chemical sensors in the human body. **TRPA1**, **TRPM8**, and **TRPV1** proteins are coexpressed in many tissues and are most frequently associated with toxicity sensing. **TRPV4** channels are cited less often, with other TRP channels (TRPC1, TRPC4, and TRPM5) being expressed to a lesser extent [29].

In summary, this special issue of Cells presents a comprehensive overview of the latest data on four TRP channel families and will hopefully convince readers of the importance of these proteins for human physiology and as drug targets for future therapeutics. 

## Figures and Tables

**Figure 1:**
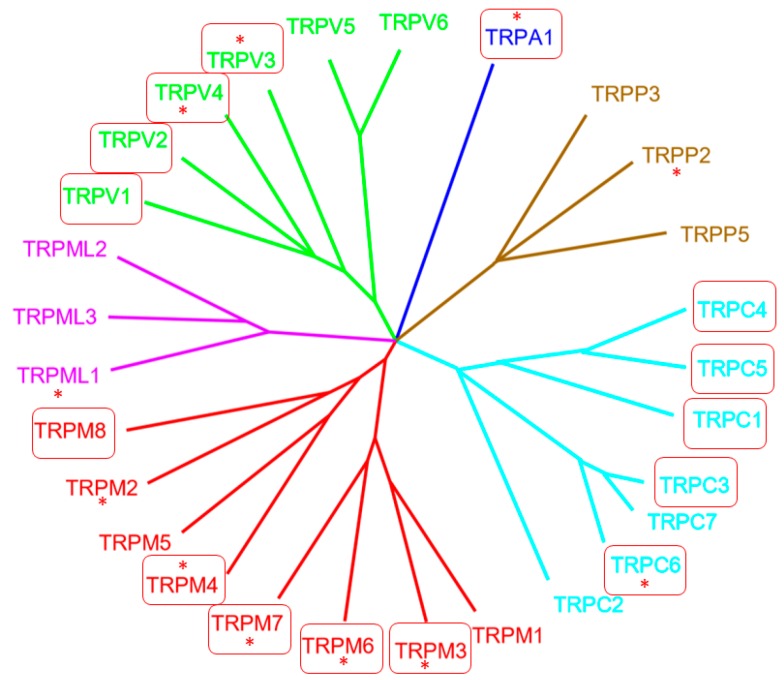
Phylogenetic tree of the transient receptor potential (TRP) superfamily in vertebrates. Boxed TRP channels are highlighted in the manuscripts of this special section. Stars (*) indicate the mutant TRP channels that cause human diseases. Picture modified from [5].
